# *TDP1* splice-site mutation causes HAP1 cell hypersensitivity to topoisomerase I inhibition

**DOI:** 10.1093/nar/gkae1163

**Published:** 2024-12-11

**Authors:** Chen Gang Goh, Aldo S Bader, Tuan-Anh Tran, Rimma Belotserkovskaya, Giuseppina D’Alessandro, Stephen P Jackson

**Affiliations:** Cancer Research UK Cambridge Institute, University of Cambridge, Cambridge, CB2 0RE, UK; Cancer Research UK Cambridge Institute, University of Cambridge, Cambridge, CB2 0RE, UK; Cancer Research UK Cambridge Institute, University of Cambridge, Cambridge, CB2 0RE, UK; Cancer Research UK Cambridge Institute, University of Cambridge, Cambridge, CB2 0RE, UK; Cancer Research UK Cambridge Institute, University of Cambridge, Cambridge, CB2 0RE, UK; Cancer Research UK Cambridge Institute, University of Cambridge, Cambridge, CB2 0RE, UK

## Abstract

HAP1 is a near-haploid human cell line commonly used for mutagenesis and genome editing studies due to its hemizygous nature. We noticed an unusual hypersensitivity of HAP1 to camptothecin, an antineoplastic drug that stabilizes topoisomerase I cleavage complexes (TOP1ccs). We have attributed this hypersensitivity to a deficiency of TDP1, a key phosphodiesterase involved in resolving abortive TOP1ccs. Through whole-exome sequencing and subsequent restoration of TDP1 protein via CRISPR-Cas9 endogenous genome editing, we demonstrate that TDP1 deficiency and camptothecin hypersensitivity in HAP1 cells are a result of a splice-site mutation (*TDP1* c.660–1G > A) that causes exon skipping and TDP1 loss of function. The lack of TDP1 in HAP1 cells should be considered when studying topoisomerase-associated DNA lesions and when generalizing mechanisms of DNA damage repair using HAP1 cells. Finally, we also report the generation of HAP1 STAR clones with restored TDP1 expression and function, which may be useful in further studies to probe cellular phenotypes relating to TOP1cc repair.

## Graphical Abstract

**Graphical Abstract:**
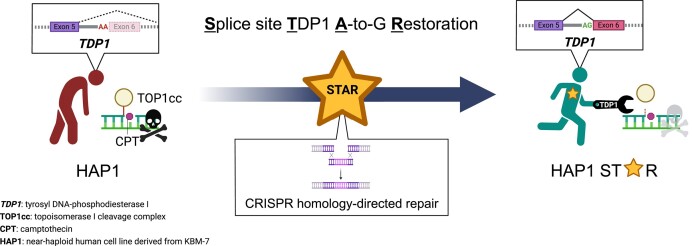

## Introduction

DNA topoisomerases regulate DNA supercoiling to facilitate essential physiological processes such as DNA replication and transcription (1,2). Topoisomerase I (TOP1) is a type IB topoisomerase that relieves torsional stress by generating a single-stranded DNA break in double-stranded DNA. This process involves the formation of a transient intermediate structure with a 5′-OH DNA end and a 3′-phosphotyrosyl covalent bond between the TOP1 catalytic tyrosine and the DNA backbone, termed the TOP1 cleavage complex (TOP1cc) (3,4). Under normal circumstances, the DNA break allows relief of DNA torsional stress via strand rotation, then the nicked DNA is religated and TOP1 is released (5,6). However, DNA lesions existing close to the TOP1cc can cause spatial misalignment between the phosphotyrosyl bond and the 5′-OH end, thereby favouring formation of a stable (abortive) TOP1cc (7). Abortive TOP1ccs create ‘roadblocks’ in the genome for transcription and replication machineries, and when replication forks encounter them, DNA nicks are converted into cytotoxic single-ended double-stranded breaks (seDSBs) (7). Camptothecin and its FDA-approved analogue, Topotecan, inhibit the religation of the nick and stabilise TOP1ccs (8,9), thereby selectively killing actively replicating and transcribing cells, including cancer cells (10,11).

To alleviate abortive TOP1cc-induced cytotoxicity, covalently attached TOP1 has to be removed to facilitate repair of the DNA lesion (7,12). Tyrosyl-DNA phosphodiesterase I (TDP1) is a key enzyme responsible for ‘detaching’ TOP1 from DNA by specifically hydrolyzing the 3′-phopshotyrosyl bonds in abortive TOP1ccs (13–15). TDP1 phosphodiesterase activity is attributed to two HKN (His-Lys-Asn) motifs that comprise crucial residues of its active site (His263, Lys265, Asn283, His493, Lys495 and Asn516 in human TDP1) (16–18). Mutations of the catalytic amino acid residues, e.g. H493R in the case of a rare neurodegenerative disease, spinocerebellar ataxia with axonal neuropathy (SCAN1) (19), lead to the formation of a steric obstacle for repair (20), camptothecin hypersensitivity (21), defective TOP1cc resolution (22) and various debilitating clinical phenotypes. Apart from TDP1 playing a predominant role in resolving persistent TOP1ccs in human cells, additional factors such as tyrosyl-DNA phosphodiesterase II (TDP2) (23,24) and the endonuclease MUS81 (25,26) have been reported to remove abortive TOP1ccs to alleviate TOP1cc-induced cytotoxicity in the absence of TDP1.

HAP1 is a near-haploid human cell line derived from KBM-7, a cell line isolated from a patient with chronic myeloid leukemia (CML) (27). The hemizygous nature of HAP1 makes it a work-horse cell line that is widely used in cell biology studies, particularly those involving precision gene engineering and genetic screens (28). Here, we report that HAP1 cells are unusually and specifically hypersensitive to camptothecin and this is underpinned by TDP1 deficiency. We demonstrate that lack of functional TDP1 in HAP1 cells is mainly attributed to a single nucleotide variant (SNV) within an intron that leads to exon(s) skipping and no detectable TDP1 protein. This work establishes the phenotypic consequences of a newly identified *TDP1* mutation, which facilitates an improved understanding of HAP1 cell lines and highlights the caveat of its utility in probing DNA damage repair (DDR) factors associated with TOP1 inhibitor treatments.

## Materials and methods

### Cell lines

HAP1 (ID: C631) cells were purchased from Horizon Discovery. HAP1 Cas9 and HAP1 Cas9 TDP2^KO^ cells lines stably expressing nuclease-active Cas9 and were generated in the lab (29). HAP1-BE3, a cell line stably expressing a human codon-optimized cytosine base editor (30), was a gift from Alberto Ciccia. HAP1-BE3 STAR clones (clone #9 and #24), where the identified *TDP1* splice variant ‘AA’ was corrected to ‘AG’, were generated via transient transfection of HAP1-BE3 cells with TrueCut Cas9 protein v2 (Thermo Fisher Scientific), a synthetic sgRNA (guide spacer: 5′-GCTTATCACCATGCACAAGC; Integrated DNA Technologies) and a HDR donor oligo repair template (5′-GCCTCGGCCTCCCAAGGTGCCGGGATTACAGGTGTGAGCCACTGAGCCTGGCCATGAATGATGTATATTAAATACTAATGCTCTCTTTTTAGGAAGAAGCCAATTCTGCTTGTGCATGGTGATAAGCGAGAGGCTAAGGCTCACCTCCATGCCCAGGCCAAGCCTTACGAGAACATCTCTCTCTGCCAGGTAAGC; Integrated DNA Technologies). Transfection was performed using 4D-Nucleofector® X unit (Lonza Biosciences) using recommended transfection conditions. The transfected cells were incubated for 2–3 days to facilitate HDR before selecting for single clones in 96-well plates via limiting dilution. For HAP1-BE3 clones without *TDP1* edit, the transfection and monoclonal selection process remained the same except without the addition of sgRNA and HDR donor oligo template.

To generate HAP1 cells complemented with human WT or A134T TDP1 under inducible expression, the cells were lentivirally transduced twice to integrate two different constructs into the genome. The first transfer plasmid construct expresses a Tet3G regulatory protein with a neomycin resistance gene (plasmid ordered from VectorBuilder) and the second construct expresses TDP1 (WT or A134T) with a puromycin resistance gene. The transfer plasmids for TDP1 (WT or A134T) expression were generated via restriction cloning using the vector plasmid (pTRE3G-HA_CMV-eGFP-T2A-puroR; custom plasmid ordered from VectorBuilder) and *TDP1* (wild type or A134T) protein coding DNA sequences (CDS from Ensembl: ENST00000335725.9 or with a mutation *TDP1* c.400G > A for *TDP1_A134T*; ordered via gene and variant synthesis from Genewiz). A transfer plasmid backbone without the TDP1 CDS (empty vector – EV) was used for transducing cells as a negative control. To generate lentiviruses carrying the above-mentioned constructs for integration, Lenti-X^TM^ 293T cells (Takara Bio) were transfected with packaging plasmids (psPAX2: Addgene #12260, pMD2.G: Addgene #12259) and the transfer plasmid using TransIT-LT1 transfection reagent (Mirus Bio). Upon each lentiviral transduction, transduced cells were selected with Geneticin^TM^ Selective Antibiotic (Thermo Fisher Scientific) or puromycin for 5 and 2 days, respectively. All versions of HAP1 cells were cultured at 37°C and 5% CO_2_ in Iscove's modified Dulbecco's medium (Sigma-Aldrich) supplemented with 10% (v/v) fetal bovine serum (FBS; BioSera), penicillin (100 U/ml), streptomycin (100 μg/ml; Sigma-Aldrich) and 2 mM L-glutamine. HAP1 cells complemented with an inducible TDP1 were cultured similarly but using Tet-negative FBS to minimize leaky expression of exogenous TDP1.

hTERT RPE-1 cell line (ID: CRL-4000; referred to as RPE-1) was purchased from ATCC. RPE-1 Cas9 and RPE-1 Cas9 TDP2^KO^ cell lines stably express nuclease-active Cas9 and were obtained within the lab (29). RPE-1 clones without TDP1 edit, which served as the control cell lines, were transfected with Cas9 protein without sgRNA and donor template. All RPE-1 cells were cultured at 37°C and 5% CO_2_ in Dulbecco's modified Eagle's medium:nutrient mixture Ham's F-12 (DMEM/F-12; Thermo Fisher Scientific), 10% (v/v) FBS (BioSera), penicillin (100 U/ml), streptomycin (100 μg/ml; Sigma-Aldrich) and 2 mM L-glutamine. U2OS cells were cultured at 37°C and 5% CO_2_ in DMEM (Thermo Fisher Scientific) supplemented with 10% (v/v) FBS (BioSera), penicillin (100 U/ml), streptomycin (100 μg/ml; Sigma-Aldrich), and 2 mM L-glutamine. All single clone verification was performed via targeted-polymerase chain reaction (PCR) utilizing Q5® High-Fidelity 2X Master Mix (New England Biolabs) followed by Sanger sequencing. The starting materials of the targeted-PCR reactions were extracted genomic DNAs either from cultured cells on a 96-well plate using QuickExtract^TM^ DNA Extraction Solution (Biosearch Technologies) or from cell pellets using Purelink^TM^ Genomic DNA Mini Kit (Thermo Fisher Scientific). Prior to Sanger sequencing, the PCR products were purified using either QIAquick PCR purification kits (Qiagen) or AMPure XP beads (Beckman Coulter) according to manufacturers’ instructions. Sanger sequencing results were subsequently analyzed on Benchling (via the sequence alignment tool) or Synthego Inference Of CRISPR Edits (ICE) to determine the editing outcomes.

### Cell viability assays

Cells were counted and plated in 96-well plates in technical duplicates and treated with indicated drugs 24 h later. These cells were then incubated in different doses of drug treatment for 3–5 days. 10% v/v (relative to the volume of culture medium) of 200 ug/ml Alamar Blue (dissolved in 1X PBS) were then added into the culture medium and mixed. The cells were incubated at 37°C and 5% CO_2_ for 3 h before measuring the fluorescence level (a proxy for cell viability; excitation wavelength: 545 nm; emission wavelength: 590 nm) on CLARIOstar microplate reader (BMG Labtech). For HAP1 cells complemented with exogenous TDP1 under inducible expression, cells were incubated in 10 ng/ml doxycycline (dox) for 48 h before plating in 96-well plates. The dox-induced TDP1 protein expression was verified by western blotting (Supplementary Figure S1C). Different numbers of cells (1000–10000) were initially plated depending on cell size, growth rate and duration of drug incubation to avoid cells becoming over-confluent before measuring the cell viability. Cells were treated with varying doses of camptothecin (C9911, Sigma-Aldrich) or etoposide (VP-16, Cayman Chemicals). All cell viability assays were performed in 3 biological replicates *n = 3*.

### RNA-seq data analyses

RNA-Seq data for HAP1 (SRR26324376 – SRR26324379), U2OS (SRR26961022 – SRR26961025) and RPE-1 (SRR21875801 – SRR21875802) cells were retrieved from the NCBI database using parallel-fastq-dump (31). The first 13 nucleotides of each read with unstable GC content were trimmed and any reads shorter than 25 nucleotides together with adapter sequences were removed using fastp (32). Quality assessment of the processed RNA-Seq data was then performed using fastqc (https://www.bioinformatics.babraham.ac.uk/projects/fastqc) and multiqc (33).

**Figure 1.**
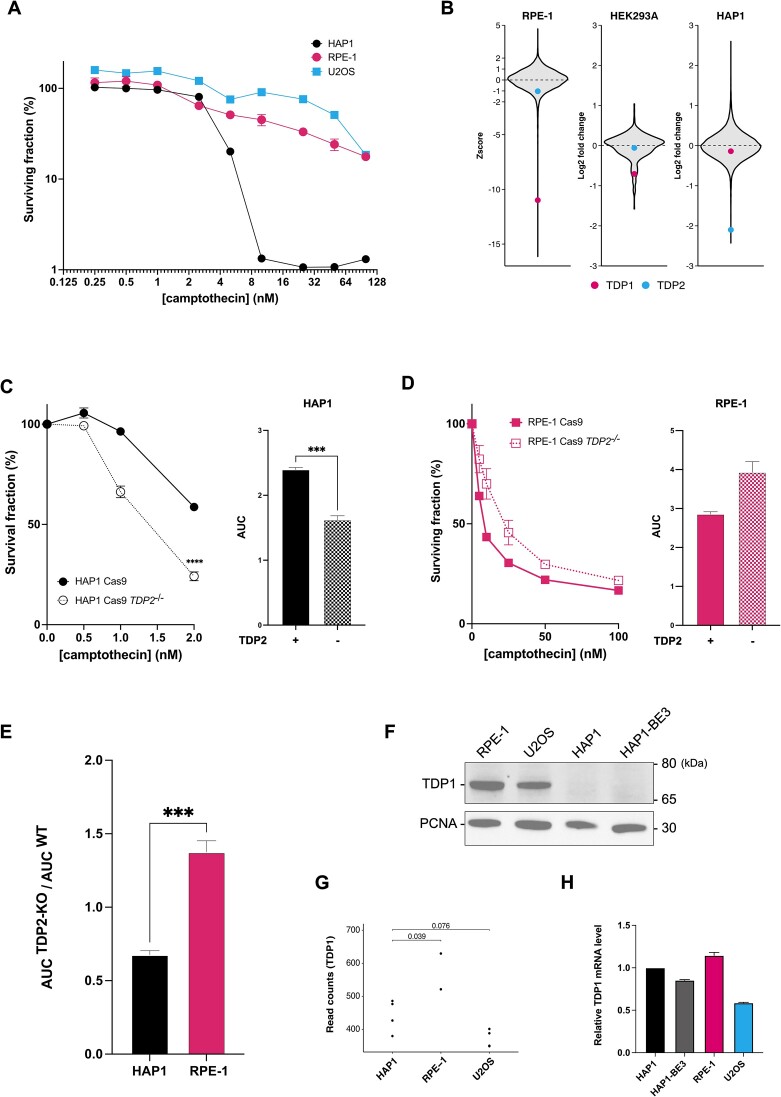
HAP1 cells are hypersensitive to camptothecin and deficient of TDP1 protein. (**A**) Cell viability dose-response curves of HAP1, human retinal pigment epithelial (RPE-1) and human osteosarcoma (U2OS) cell lines to camptothecin treatment. (**B**) Chemogenetic CRISPR-Cas9 knockout screens in RPE-1 (49), HEK293A (48) and HAP1 (47) cells subjected to camptothecin treatment, highlighting the relative impacts on gRNAs targeting *TDP1* (•) and *TDP2* (•) in the cell populations interpreted as Zscore (54) or log2 fold-change. (**C, *left***) Cell viability dose-response curves to camptothecin of HAP1 and (**D, *left***) RPE-1 cells in the presence or absence of TDP2. For the comparison in (C, *left*) at 2 nM camptothecin, the *P-value* (**** *P*< 0.0001) was calculated using two-way ANOVA. (**C & D, *right***) Quantified area under curve (AUC) data from the corresponding dose-response curves. In (**A**), (**C**) & (**D**), 5000 HAP1 cells and 1000 RPE-1 and U2OS cells were seeded in technical duplicates 24 h before camptothecin treatment. Treated cells were incubated for 4 days before measuring the cell viability with Alamar Blue fluorescent indicator. Experimental data were the average of 3 biological repeats ± s.e.m. (**E**) Comparison of the differential AUCs (AUC^TDP2-KO^/AUC^WT^) between (**C**) HAP1 and (**D**) RPE-1 cells. The *P-value* (*** *P*< 0.001) was calculated through an unpaired two-tailed *t*-test (i.e. one-way ANOVA). (**F**) Total cell lysates of individual cell lines were immunoblotted for TDP1 protein. PCNA serves as a loading control. The sizes (kDa) of reference proteins in PageRuler prestained protein ladder are indicated on the right. (**G**) Normalized read counts of *TDP1* mRNA in HAP1, RPE-1 and U2OS cell lines. RNA read counts for each cell line were sourced from RNA-Seq data available online and analyzed using DESeq2. Each column represents an RNA-Seq experiment with multiple biological replicates (black dots, *n = 2–4*). Adjusted *p-values* of *TDP1* RNA read count comparison against HAP1 cells are shown. (**H**) Comparison of *TDP1* mRNA level (normalized against PUM1 as a stable housekeeping gene) between different cell lines via RT-qPCR. Normalized *TDP1* mRNA levels were compared against HAP1 cells. Experimental data were the average of three technical replicates ± S.E.M.

To compare *TDP1* gene expression (RNA level) between different cell lines, the processed data was first mapped onto the human genome (GRCh38.p14, Ensembl release 110) with Salmon (34). The mapped reads were then imported to R using GenomicFeatures (35) and tximport (36). Differential analysis was performed with DESeq2 package (37) to compare the *TDP1* expression levels between HAP1, U2OS and RPE-1 cells. Normalized read counts of *TDP1* mRNA in different cell lines were plotted (Figure 1G) using ggplot2 (38).

**Figure 2.**
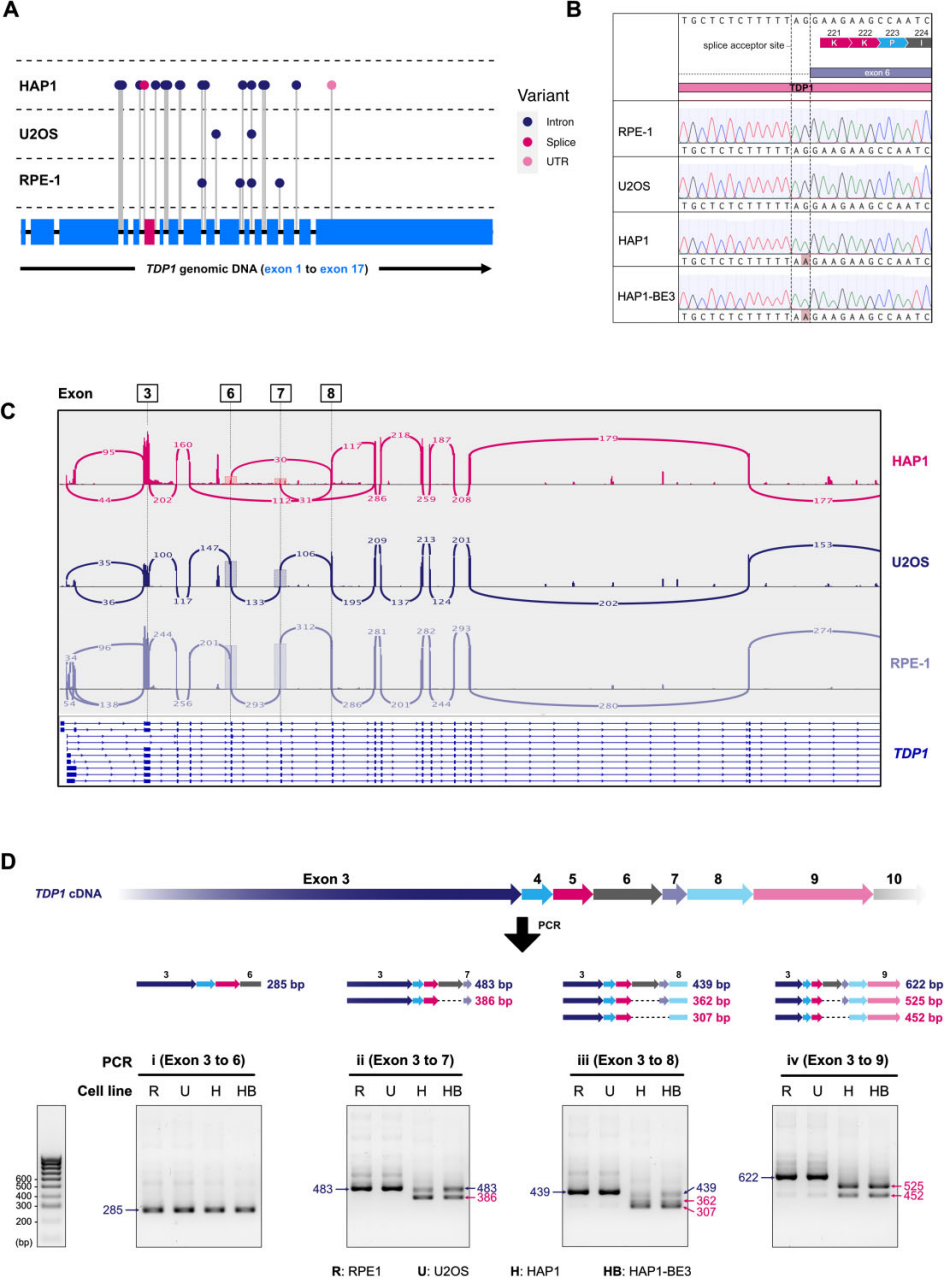
*TDP1* splice variant impairs *TDP1* RNA splicing and causes exon skipping in HAP1 cells. (**A**) Pairwise comparison of the sequenced non-coding DNA (intronic regions adjacent to the exon ends) of *TDP1* in HAP1, U2OS and RPE-1 cells against the reference genome. Introns have been compressed into fixed lengths (50 bp width; black connecting lines) to showcase the entire *TDP1* gene, where the blue horizontal rectangles are individual exons except for exon 6 (magenta rectangle). Genomic variants in the *TDP1* non-coding sequences for individual cell lines are highlighted as a ‘lollipop’ and coloured according to the variant type: (•) variant in the **intron**, (•) variant that affects **splice** donor/acceptor site, (•) variant in the 5′ or 3′ untranslated region according to the whole-exome sequencing. (**B**) Sanger sequencing chromatograms of RPE-1, U2OS, HAP1 and HAP1-BE3 cells showing the genomic sequence of *TDP1* around exon 6. The *TDP1* c.660–1G > A splice-site mutation (‘AA’ splice acceptor site) is unique to HAP1 and HAP1-BE3 cells. (**C**) Sashimi plots of TDP1 RNA expression in HAP1 (top), U2OS (middle) and RPE-1 (bottom) cells where the peaks indicate the RNA coverage at the coding sequence of different exons and the peak height reflects the depth of mapped reads from RNA-Seq results. The peaks at exons 3, 6, 7 and 8 were annotated and the dotted line boxes highlight the peak heights of exons 6 and 7 in HAP1, U2OS and RPE-1 cell lines. (**D**) *Top*: Schematics of the expected PCR amplicon sizes (in bp) of multiple targeted PCRs – i, ii, iii and iv (with reverse primers annealing to exon 6, 7, 8 or 9 respectively) of the *TDP1* cDNA, taking into account when exon 6 or exon 6 + 7 skipping occur. Note: forward primers annealing to exon 3 are not identical in different PCRs. *Bottom*: Separation of DNA products amplified using reverse transcribed *TDP1* cDNA in different cell lines (**R**: RPE-1, **U**: U2OS, **H**: HAP1, **HB**: HAP1-BE3) on a 2% agarose gel to test for *TDP1* mRNA length and purity. The estimated size (bp) of DNA bands for each PCR (i, ii, iii and iv) is annotated (smaller PCR products due to exon skipping are annotated in magenta). The DNA ladder (bottom left) is made up of DNA bands at 100 base-pair intervals.

To determine whether exon skipping is occurring in TDP1 mRNA in HAP1 cells, the sequenced reads of the processed RNA-Seq data were mapped onto the human genome using hisat2 (39). The mapped reads were sorted and indexed with SAMtools (40) before importing them to IGV (41) for sashimi plot generation (Figure 2C). For a clearer visualization, splice junctions with less than 20 mapped reads were excluded to only show the predominant splicing junctions.

### RNA extraction, RT-qPCR, and PCRs

Total RNAs were extracted from cells using the RNeasy Mini Kit (Qiagen) with the inclusion of the optional step of RNase-free DNase-I (Qiagen) according to manufacturer's instructions. For RT-qPCR, cDNAs were generated from 1 ug of extracted total RNA using SuperScript^TM^ IV VILO Master Mix (Thermo Fisher Scientific) according to manufacturer's instructions. For each qPCR reaction (20 ul), 0.5 ul of the reverse transcribed cDNAs was included in a mixture consisting of a qPCR primer pair and Fast SYBR^TM^ Green Master Mix (Thermo Fisher Scientific). To compare TDP1 mRNA expression between different cell lines, a stable housekeeping gene *PUM1* was used to normalize the TDP1 mRNA abundance. The qPCR primers are as follow: *PUM1* (F: 5′-CGGGAGATTGCTGGACATATAA; R: 5′-CACATCCACCATGAGTTGGTA) and *TDP1* (F: 5′-AATGTGCGGACCAGTTTAG; R: 5′-AAGTCTCAGCTGACCATTTG). qPCR reactions were prepared in technical triplicates.

To test the exon skipping, TDP1 mRNA was reverse transcribed with a TDP1 gene-specific primer (5′-ACATGTTCCCATGCGTATCC) and the SuperScript^TM^ IV Reverse Transcriptase (Thermo Fisher Scientific). The cDNA was then used as a template for the first round of PCR, where almost the entire TDP1 cDNA was amplified, followed by a second round of PCR that amplified the section around exon 6 in the cDNA. In the second round of PCR, four PCRs involving four primer pairs tiling across different exons were carried out. The primer pairs amplified TDP1 cDNA between exon 3 and exons 6, 7, 8 or 9, in which the products were then separated on a 2% agarose gel. The presence of DNA bands shorter than the expected amplicon lengths suggested the presence of exon skipping in TDP1 mRNA. The first round of PCR amplified full length TDP1 cDNA copies, performed using the following primer pair: F: 5′-ATGTCTCAGGAAGGCGATTATG; R: 5′-ACATGTTCCCATGCGTATCC. The primer pairs of the second round of PCRs are as follows: exons 3–6 (F: 5′-GGAAGACGAGTATGAGACATCAG-3′; R: 5′-GCCTTAGCCTCTCGCTTATC), exons 3–7 (F: 5′-AGAAGCAGGCTGAGAAAGTG; R: 5′-GGTGTGTTCCAAACGCAATATC), exons 3–8 (F: 5′-AGAGGAGGAAGACGAGTATGAG; R: 5′-GATGAGGTTGGAGGTGTGTATG), exons 3–9 (F: 5′-AGAAGCAGGCTGAGAAAGTG; R: 5′-AGAGAGATCGTGCTTGTGAATG).

### Immunoblotting

Cells were lysed in protein extraction lysis buffer [50 mM Tris-HCl pH7.5, 2% SDS, serine/threonine phosphatase inhibitor cocktail (Sigma-Aldrich) and protease inhibitor cocktail (Roche)] and incubated for 5 min at 95°C. Proteins in cell lysates were then resolved in NuPAGE^TM^ 4–12% Bis-Tris precast gels (Thermo Fisher Scientific). After transferring onto nitrocellulose membranes, the membranes were blocked with blocking buffer [5% bovine serum albumin in TBS-T (tris-buffered saline with Tween 20, 0.1%)] and incubated with primary antibodies diluted in blocking buffer. The membranes were subsequently washed with TBS-T before probing with secondary antibodies in TBS-T. Membranes were then washed again with TBS-T and incubated with SuperSignal West Pico PLUS (enhanced chemiluminescence; Thermo Fisher Scientific) according to manufacturer's instructions. Chemiluminescence of probed proteins was visualized on a ChemiDoc imaging system (Bio-Rad). Antibodies used were: TDP1 (Bethyl Lab Inc., A301-618A; 1:1000), PCNA (Santa Cruz Biotechnology, sc56; 1:1000) and vinculin (Abcam, ab219649; 1:1000).

To test for TDP1 protein stability, HAP1 cells complemented with either WT or A134T TDP1 under an inducible promoter, were cultured in 10 ng/ml of dox for 24 h and removed thereafter. The cells were further incubated up to 30 h in the absence of dox. Cells were harvested at different time points (8, 24 and 30 h) upon dox removal. Cell lysates were prepared and TDP1 protein was immunoblotted in a similar workflow as above.

### Whole-exome sequencing and data analyses

HAP1, RPE-1 or U2OS cells were seeded on 10 cm dishes for 48 h. To harvest, cells were washed once in 1X PBS, incubated with cytoplasmic lysis buffer (50 mM HEPES pH7.9, 10 mM KCl_2_, 1.5 mM MgCl_2_, 0.34 M sucrose, 0.5% Triton X-100, 10% glycerol, 1 mM DTT and 0.1mg/mL RNase A) for 10 min at room temperature, washed once in cytoplasmic lysis buffer and finally scraped in genomic extraction buffer (50mM Tris pH 8.0, 5mM EDTA, 1% SDS, 0.5mg/mL proteinase K (Thermo Fisher Scientific, 25 530 049). Lysates were transferred to 2 ml DNA LoBind microcentrifuge tubes (Thermo Fisher Scientific, 0 030 108 426) and incubated at 60°C with 500 round per minute (rpm) agitation for 1 h. Sodium acetate pH 5.2 was added to a final concentration of 300mM and 2.5 volumes of 100% ethanol was added. Samples were incubated on ice for 1 h to precipitate the genomic DNA, which was then pelleted at 21 000 g for 20 min at 4°C. Genomic DNA pellets were washed once in 75% ethanol and re-pelleted for 10 min before being allowed to air-dry followed by resuspension in nuclease-free water.

DNA samples were diluted to 100 ng/μl in 300 μl in 1.5 ml Bioruptor Plus TPX Microtubes (Diagenode, C30010010-300) and sonicated in a Bioruptor Plus sonicator for 20 cycles of 30 s on/30 s off with active chilling at 4°C, shearing the DNA to an average length of ∼600–800 bp. 500 ng of sheared genomic DNA was used for library preparation using the IDT xGen DNA library preparation kit (Integrated DNA Technologies, at 10 009 861) coupled to the xGen Exome Hyb Panel v2 for exome enrichment (Integrated DNA Technologies, 10 005 151) all according to the manufacturer's instructions. Libraries were then pooled at equimolar concentrations and sequenced on an Illumina NovaSeq 6000 with paired-end 150 cycles.

Raw fastq files (deposited in ArrayExpress under accession number E-MTAB-14219) were filtered using fastp (32) and aligned to the hg38 human reference genome using Bowtie2 (42). Variant calling was conducted initially using Manta (43) to identify structural variants and candidate indels, and Strelka2 (44) was subsequently used to identify indels and base substitutions. All analysis scripts with exact parameters are available at https://github.com/aldob/wes_variant_analysis.

### Alkaline comet assays

HAP1 cells were grown to ∼80% confluency and the adherent cells were treated with camptothecin or hydrogen peroxide for 1 h. After treatment, cells were rinsed once with ice cold 1X PBS (without CaCl_2_ and MgCl_2_) before being detached with trypsin. Detached cells were subsequently resuspended in the same ice cold 1X PBS and their cell densities were determined. Each cell suspension was then diluted to ∼200000 cells/ml and chilled on ice. Cell suspensions were then mixed with CometAssay® LMAgarose (R&D Systems) at 1:10 ratio before being spread on the 2-well CometSlides (R&D Systems). Cells on the slides were prepared and subjected to electrophoresis in an alkaline condition with the CometAssay® Electrophoresis System II according to manufacturer's recommendations. After electrophoresis, SYBR Gold-stained ‘comets’ on the slides were imaged with an Opera Phenix^TM^ Plus (Revvity) at 10× magnification. Images were then analyzed by using OpenComet (45) to quantify Olive tail moment (46) to determine the extent of DNA single-stranded breaks.

## Results

### HAP1 cells are hypersensitive to camptothecin and deficient in TDP1

Despite their great utility in genome editing, HAP1 cells display an unusual hypersensitivity to camptothecin relative to other human cell lines (Figure 1A). To gain further insights into why HAP1 cells are distinctively sensitive to camptothecin, we compared publicly available data from CRISPR gene-knockout screens with camptothecin in HAP1 cells (47) and other human cell lines (48,49) (Figure 1B). As expected, we observed *TDP1* appearing as a hypersensitivity hit (negative log2 fold-change or Zscore values) in the screens for human cell lines HEK293A and RPE-1, as TDP1 is crucial for resolving camptothecin-induced abortive TOP1ccs. In other words, losing TDP1 confers increased sensitivity towards camptothecin because TOP1ccs persist, leading to cytotoxicity (21,50,51). In contrast, screen outputs indicate that losing TDP1 in HAP1 cells does not confer further sensitivity to camptothecin. Conversely, the screen data indicate that loss of TDP2 heightens camptothecin sensitivity in HAP1 cells but not in HEK293A and RPE-1 cells. These data suggested that HAP1 cells might be deficient in TDP1 and so rely on TDP2 to partially compensate for its function in resolving abortive TOP1ccs (23,24). To test this, we next compared camptothecin sensitivities of HAP1 and RPE-1 cells that had or had not been subjected to CRISPR-Cas9 genome engineering to knock out *TDP2* (*TDP2*^KO^) (29) (Figure 1C–E). Confirming our interpretations of the screen results, knocking out *TDP2* enhanced the camptothecin hypersensitivity of HAP1 cells (Figure 1C), but not in RPE-1 cells (Figure 1D).

Based on the above data, we hypothesized that HAP1 cells might be defective in TDP1. Thus, we immunoblotted whole cell extracts of HAP1, RPE-1 and U2OS cells and observed that TDP1 protein was detected in RPE-1 and U2OS cells extracts but not in extracts of HAP1 cells – both a commercial HAP1 line (Horizon Discovery) and a HAP1 clone, HAP1-BE3 stably expressing a cytidine base editor obtained from another laboratory (52) (Figure 1F). Furthermore, we analyzed publicly available RNA-Seq data to determine whether the TDP1 protein deficiency in HAP1 cells stems from a reduced transcript abundance. When comparing TDP1 mRNA levels of HAP1, RPE-1 and U2OS cells, we observed similar *TDP1* read counts in HAP1 and U2OS cells and higher read counts in RPE-1 cells (Figure 1G). To extend these observations, we employed quantitative polymerase chain reactions (qPCRs) performed on reverse transcribed *TDP1* cDNA normalizing against the housekeeping gene, *PUM1*, with consistent expression across different cell lines (53). The normalized TDP1 RNA level was highest in RPE-1, followed by HAP1 then U2OS (Figure 1H), in line with the RNA-seq data. We concluded that the apparent absence of TDP1 protein in HAP1 cells is unlikely to reflect reduced *TDP1* transcription or mRNA stability.

### TDP1_A134T variant does not affect camptothecin sensitivity or protein stability

To investigate whether the *TDP1* gene in HAP1 cells carries a mutation(s) that could potentially impact protein levels, we undertook whole-exome sequencing of HAP1 cells and inspected *TDP1* exon sequences initially for any variants that could result in a non-synonymous mutation. Comparison of the *TDP1* exon sequences in HAP1 cells with the human reference genome revealed a unique SNV of *TDP1* c.400G > A (TDP1 p.Ala134Thr; Supplementary Figure S1A) reported to be associated with spinocerebellar ataxia, autosomal recessive, with axonal neuropathy 1 (SCAN1; RCV000282718.5 on ClinVar). Despite being classified as a benign variant, we tested whether A134T TDP1 affects cellular resistance towards camptothecin by expressing inducible wild type (WT) or A134T TDP1 in HAP1 cells and performing cell viability assays under various camptothecin doses. We observed that, compared to control cells, expression of WT or A134T TDP1 in HAP1 cells conferred equivalent degrees of camptothecin resistance (Supplementary Figure S1B, C). Additionally, no difference in protein stability between WT and A134T TDP1 was detected when we compared their expression levels by western blotting for up to 30 h without doxycycline (dox) after 24 h of initial induction (Supplementary Figure S1D). These data suggested that the A134T TDP1 variant has neither impaired function nor reduced stability, in line with its benign classification on ClinVar.

### TDP1 splice-site mutation impairs mRNA splicing and renders HAP1 cells TDP1 deficient

Considering that A134T TDP1 (the only non-synonymous variant) does not contribute to TDP1 deficiency in HAP1 cells, we then checked for variants in non-coding regions adjacent to TDP1 exons, picked up by our sequencing approach (Figure 2A). In particular, the HAP1 *TDP1* c.660–1G > A splice variant (Figure 2A) stood out to us and was verified via Sanger sequencing in both HAP1 and HAP1-BE3 cells (Figure 2B). This SNV abrogates the canonical splice acceptor site ‘AG’ (‘AA’ in HAP1 cells) upstream of exon 6 and is predicted to disrupt mRNA splicing resulting in downstream exon(s) skipping and/or intron retention. The conservation of the ‘AG’ splice acceptor site in *TDP1* across 10 different vertebrate species further underscores the likely detrimental implications of disrupting it (Supplementary Figure S2A).

To check for any mRNA splicing defect of *TDP1* in HAP1 cells, we aligned and mapped RNA reads of *TDP1* in HAP1, RPE-1 and U2OS cell lines (derived from RNA-Seq data available on NCBI) onto the human reference genome to create a sashimi plot. A sashimi plot shows RNA read coverages at exons as peaks, where the peak height reflects the depth of mapped reads and each arc connecting two peaks indicates splicing of the two exons. In HAP1 cells, relative to other exons, very few RNA reads aligned to both exons 6 and 7. RNA read depth at exon 8 was also significantly lower. Moreover, minimal intron inclusion was observed between exons 5 and 8 (Figure 2C). These data pointed toward a faulty splicing event for *TDP1* mRNA in HAP1 cells.

To further explore *TDP1* exon skipping in HAP1 cells, we compared the lengths of regions of the spliced mRNA in HAP1 against those in RPE-1 and U2OS cells. Towards this purpose, we generated *TDP1* cDNAs from each cell line through reverse transcription and used this as a starting template for PCRs. Four pairs of PCR primers were used to anneal to exons 3, 6, 7, 8 and 9. Should there be any exon skipping of exons 6, 7 or 8, PCR amplicons shorter than the expected size would be observed. In the absence of an RNA splicing defect in the control cell lines – RPE-1 (R) and U2OS (U) – relatively clean bands were observed in PCRs amplifying exons 3–6 (i), 3–7 (ii), 3–8 (iii) and 3–9 (iv), with amplicon sizes that corresponded to correctly spliced *TDP1* mRNA. HAP1 (H) and HAP1-BE3 (HB) cells showed amplicons of equal length in PCR i (across exons 3–6) compared to control cells. However, shorter amplicons in PCRs ii, iii, iv (across exons 3–7, 3–8, 3–9, respectively) were present in HAP1 and HAP1-BE3 cells compared to the controls (Figure 2D).

The above findings indicated that the exon skipping event in HAP1 cells occurs only for exon 6 and beyond, in line with our observations from the sashimi plots. We estimated the size of the shorter amplicons in PCRs ii, iii and iv (Figure 2D) and combined our observations from the sashimi plots to establish that *TDP1* mRNAs in HAP1 cells predominantly: (1) skip exon 6 only; or (2) skip exons 6 & 7. Exon skipping outcome (1) would lead to a frameshift mutation and a premature stop codon in exon 8 (out of 17 exons in total; Supplementary Figure S2B). Such a mRNA product would likely be degraded through nonsense-mediated decay (55,56). On the other hand, exon skipping outcome (2) would give rise to an in-frame mutation leading to a truncated protein lacking amino acid residues 253–264 (Supplementary Figure S2B), including the catalytic residue H263 that mediates the first step of 3′-phosphotyrosyl bond cleavage (17,57). Considering the presence of TDP1 transcripts in HAP1 cells (Figure 1F, G), the absence of a detectable HAP1-exclusive truncated TDP1 protein on the western blot (Supplementary Figure S2C) suggested that TDP1 might have impaired protein folding and stability upon loss of catalytic residues in exon 7 and 8. Whatever the case, we deduce that any of the two exon skipping outcomes caused by the *TDP1* c.660–1G > A variant (thereafter referred as splice-site mutation) would not give a functional TDP1 protein in HAP1 cells. These data suggested that the camptothecin sensitivity and TDP1 deficiency in HAP1 cells is likely attributed to the *TDP1* splice-site mutation that we have identified.

### Correcting TDP1 splice-site mutation restores TDP1 protein expression and function in HAP1 cells

**Figure 3.**
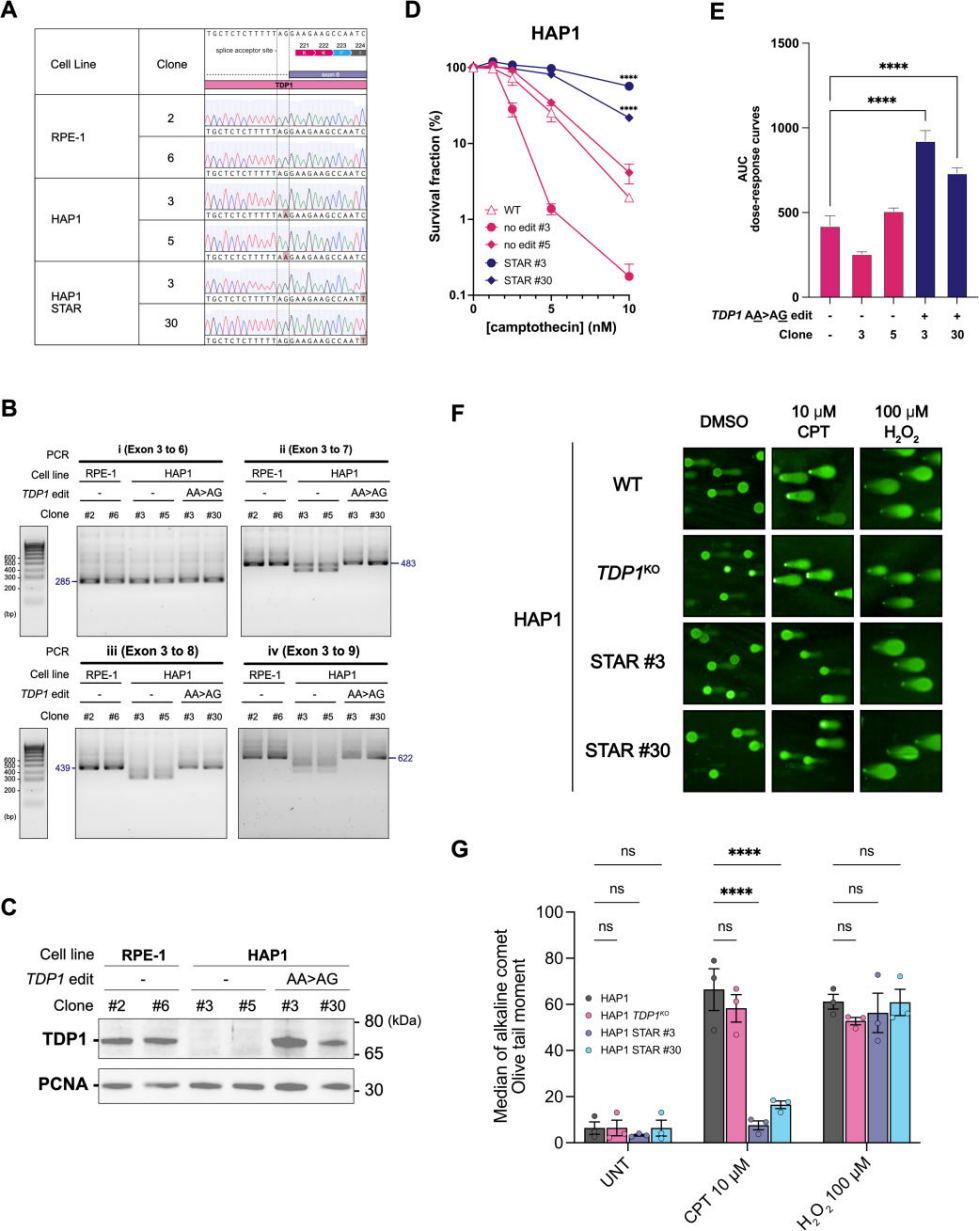
Correcting *TDP1* splice-site mutation restores TDP1 protein expression and function in HAP1 cells. (**A**) Sanger sequencing chromatograms of two clones each from RPE-1, HAP1 and HAP1 STAR cells, where the HAP1 STAR clones had had their endogenous *TDP1* splice acceptor mutant ‘AA’ successfully edited into functional ‘AG’ via DNA-templated HDR. Two clones (HAP1 STAR Clone #3 & #30) were identified. A silent mutation of *TDP1* c.672C > T was also observed in the edited HAP1 STAR clones. This mutation was intentionally included in the editing template to minimize re-annealing of gRNA to the target DNA sequence upon successful genome editing. (**B**) Separation of DNA products amplified using reverse transcribed *TDP1* cDNA in edited (HAP1 STAR) and unedited HAP1 clones as well as in RPE-1 clones on a 2% agarose gel to test for *TDP1* mRNA length and purity. The estimated size (bp) of DNA bands without exon skipping (full length of mRNA) for each PCR (i, ii, iii and iv) is annotated. See top part of Figure 2D. The presence of any DNA bands of smaller sizes demonstrate the phenomenon of exon skipping in *TDP1* mRNA. The DNA ladder (left) is made up of DNA bands at 100 base-pair intervals. (**C**) Total cell lysates of HAP1 STAR and other unedited clones (HAP1 and RPE-1) were immunoblotted to detect the recovery of TDP1 protein expression upon editing. PCNA serves as a loading control. (**D**) Cell viability dose-response curves to camptothecin of HAP1 STAR clones relative to unedited HAP1 clones. Around 10 000 HAP1 cells were seeded in technical duplicates 24 h before camptothecin treatment. Treated cells were incubated for 3 days before measuring the cell viability with Alamar Blue fluorescent indicator. Experimental data were the average of three biological repeats ± S.E.M. For the comparison against ‘WT’ HAP1 at 10 nM camptothecin, the *P-value* (**** *P*< 0.0001) was calculated using two-way ANOVA. (**E**) Quantified AUC based on the cell viability dose-response curves in D. The *P-value* (**** *P*< 0.0001) was calculated through ordinary one-way analysis of variance (ANOVA). Despite showing only the comparison of HAP1 STAR clones #3 and #30 against the unedited HAP1 polyclonal cells, their comparison against unedited HAP1 clones (#3 and #5) were also significant with *P-value*≤ 0.0001. (**F**) Representative images of alkaline ‘comets’ for ‘WT’ HAP1, HAP1 *TDP1*^KO^, HAP1 STAR #3, and HAP1 STAR #30 upon different treatment conditions (UNT: untreated, CPT: camptothecin, H_2_O_2_: hydrogen peroxide). H_2_O_2_ (100 μM) treatment was included as a positive control for DNA single-stranded break induction. (**G**) Quantified Olive tail moment of the ‘comets’, which reflects the amount of DNA single-stranded breaks. Experimental data were the mean (bar height) of the population medians in 3 biological repeats (dots) ± S.E.M. 82–150 comets were analyzed for each condition in each repeat. Different cell lines were compared against ‘WT’ HAP1 in each treatment condition using ordinary two-way ANOVA with the following reported *P-values*: (ns) *P*> 0.05 and (****) *P*< 0.0001.

To confirm that TDP1 deficiency in HAP1 cells is caused by the splice-site mutation, we sought to reverse the endogenous *TDP1* splice-site mutation to restore the splice acceptor site (edit AA to AG) via a Cas9-mediated homology-directed repair (HDR) genome editing approach. Thus, we transfected HAP1 and HAP1-BE3 cells with nuclease-active Cas9, a guide RNA that targets the locus around the splice acceptor site and a DNA repair template consisting of a DNA sequence with the desired, mutation-correcting repair outcome. Upon screening the edited cell populations, we successfully identified *TDP1*-edited clones of HAP1 (Figure 3A) and HAP1-BE3 (Supplementary Figure S3A) via Sanger sequencing (thereafter referred to as STAR [**S**plice-site **T**DP1 **A**-to-G **R**estoration] versions of HAP1 cells). *TDP1* RNAs of STAR clones (HAP1: #3 and #30; HAP1-BE3: #9 and #24) were reverse transcribed and PCR-amplified before separating the PCR amplicons on a 2% agarose gel to determine whether exon skipping in the *TDP1* mRNA had been corrected. RPE-1 and unedited HAP1 clones were tested simultaneously as controls. In each of the four PCRs (i, ii, iii, iv), we observed only one predominant DNA band without exon skipping, for all mutation-corrected HAP1 and HAP1-BE3 STAR clones tested, where the DNA band patterns were essentially identical to those from RPE-1 cells (Figure 3B; Supplementary Figure S3B).

The above findings supported the notion that the *TDP1* splice-site mutation is indeed responsible for the defective RNA splicing event of the *TDP1* mRNA in HAP1 cells. To further explore this, we immunoblotted whole cell extracts of HAP1 and HAP1-BE3 STAR clones with anti-TDP1 antibody. Strikingly, this demonstrated a recovery of TDP1 protein expression relative to the unedited clones (Figure 3C; Supplementary Figure S3C). Moreover, through cell viability assays, we established that restoration of TDP1 protein levels in edited HAP1 and HAP1-BE3 STAR clones was associated with greater camptothecin resistance relative to their unedited counterparts (Figure 3D, E; Supplementary Figure S3D, E). This implied that HAP1 STAR cells possessed an improved repair capacity for camptothecin-induced abortive TOP1ccs due to a greater availability of functional TDP1. To examine this more directly, we treated HAP1 cells and HAP1 STAR clones with varying concentrations of camptothecin for 1 h and performed alkaline single cell gel electrophoresis assays (i.e. alkaline comet assays). Strikingly, upon camptothecin treatment, HAP1 STAR clones exhibited shorter Olive tail moment (46) (smaller comet tail:head ratio; Figure 3F, G and Supplementary Figure S4) than ‘wild type (WT)’ HAP1 and HAP1 *TDP1*^KO^ cells. By contrast, no significant differences were observed in their Olive tail moments produced by hydrogen peroxide treatment. We conclude that the differences observed upon camptothecin treatment are attributed to HAP1 STAR cells possessing functional TDP1 to resolve camptothecin-induced abortive TOP1ccs effectively, and therefore minimizing DNA single-stranded breaks as detected in alkaline comet assay conditions. Combining the above observations, we conclude that the *TDP1* c.660–1G > A splice-site mutation causes the deficiency of functional TDP1 in HAP1 cells and explains why, compared to other human cell lines, HAP1 cells are unusually sensitive to camptothecin treatment.

## Discussion

In summary, we have explored the unusual hypersensitivity of HAP1 cells to camptothecin treatment and have discovered that the key factor behind this phenomenon is the lack of functional TDP1. Through whole-exome sequencing, we identified a *TDP1* splice-site mutation in HAP1 cells that leads to loss of the splice acceptor site upstream of *TDP1* exon 6. We showed, using both RNA-seq data and targeted PCRs of *TDP1* cDNA, that the *TDP1* splice-site mutation results in exon(s) skipping. Considering no significant reduction in *TDP1* transcript level in HAP1 cells compared to other cells (Figure 1G-H), we postulate that the loss of amino acid residues at the active site encoded by the skipped exon 7 leads to TDP1 protein misfolding, instability and its consequential deficiency as observed by western blotting. This cause-and-effect relationship between the *TDP1* splice-site mutation and TDP1 protein deficiency was substantiated by reverting the endogenous *TDP1* splice-site mutation in both HAP1 and HAP1-BE3 cells. In STAR clones where the *TDP1* splice-site mutation was reversed, we observed restoration of faithful RNA splicing (Figure 3B; Supplementary Figure S3B) and recovery of TDP1 protein expression and function in imparting camptothecin resistance.

Our discovery of TDP1 deficiency in HAP1 cells is important for studies generalizing mechanisms of DDR using HAP1 cells. As one of the most widely utilized cell models in high-throughput CRISPR-Cas9-mediated screens (28,52,58–61), one must consider the possibility that DDR networks in HAP1 cells might have adapted over time to some degree to accommodate for the absence of functional TDP1. This is, in our opinion, likely to be the case because TDP1 is considered a non-essential gene and there exist overlapping repair factors/pathways that promote removal of abortive TOP1ccs (62,63).

Interestingly, we also identified the *TDP1* splice variant in the HAP1 parental cell line, KBM7 (Supplementary Figure S5A), which was derived from a patient with CML. We subsequently looked into the Genomics Drug Sensitivity in Cancer database and noticed that the half maximal inhibitory concentrations (IC50s) for camptothecin treatment are unusually low across many blood cancer cell lines (Supplementary Figure S5B, C). Furthermore, adult T cell leukemia (ATL) cells were separately reported to suffer from reduced TDP1 expression (64). While it remains to be validated whether the increased camptothecin sensitivity of blood cancer cell lines is actually caused by TDP1 deficiencies, these observations suggest opportunities for using TOP1 poisons to better treat certain blood cancers.

Lastly, we have also generated HAP1 STAR clones with restored functional TDP1 protein, which may be useful in future studies to explore TOP1 biology and broader DDR mechanisms. Additionally, the HAP1-BE3 STAR clones we generated, which possess a haploid genome and stably express a cytosine base editor, could be utilized for base editing to efficiently probe phenotypes of variants of TDP1 and other factors involved in repairing abortive TOP1ccs.

## Supplementary Material

gkae1163_Supplemental_File

## Data Availability

The data relevant to this article are presented or cited in the article, in its online supplementary material and from the corresponding author RNA-Seq data accession numbers: SRR26324376 - SRR26324379, SRR26961022 - SRR26961025, SRR21875801- SRR21875802 (https://www.ncbi.nlm.nih.gov/sra) Whole-exome sequencing ArrayExpress accession number: E-MTAB-14219 (https://www.ebi.ac.uk/biostudies/arrayexpress/studies/E-MTAB-14219) Whole-exome sequencing analysis script and parameters: https://github.com/aldob/wes_variant_analysis and https://zenodo.org/doi/10.5281/zenodo.12635386.
