# Supplementary material for: TDP1 splice-site mutation causes HAP1 cell hypersensitivity to topoisomerase I inhibition
Source: Nucleic Acids Res. 2024 Dec 11;53(2):gkae1163. doi: 10.1093/nar/gkae1163 (PMC11754736; doi:10.1093/nar/gkae1163)
Supplement: gkae1163_Supplemental_File [file gkae1163_supplemental_file.pdf]

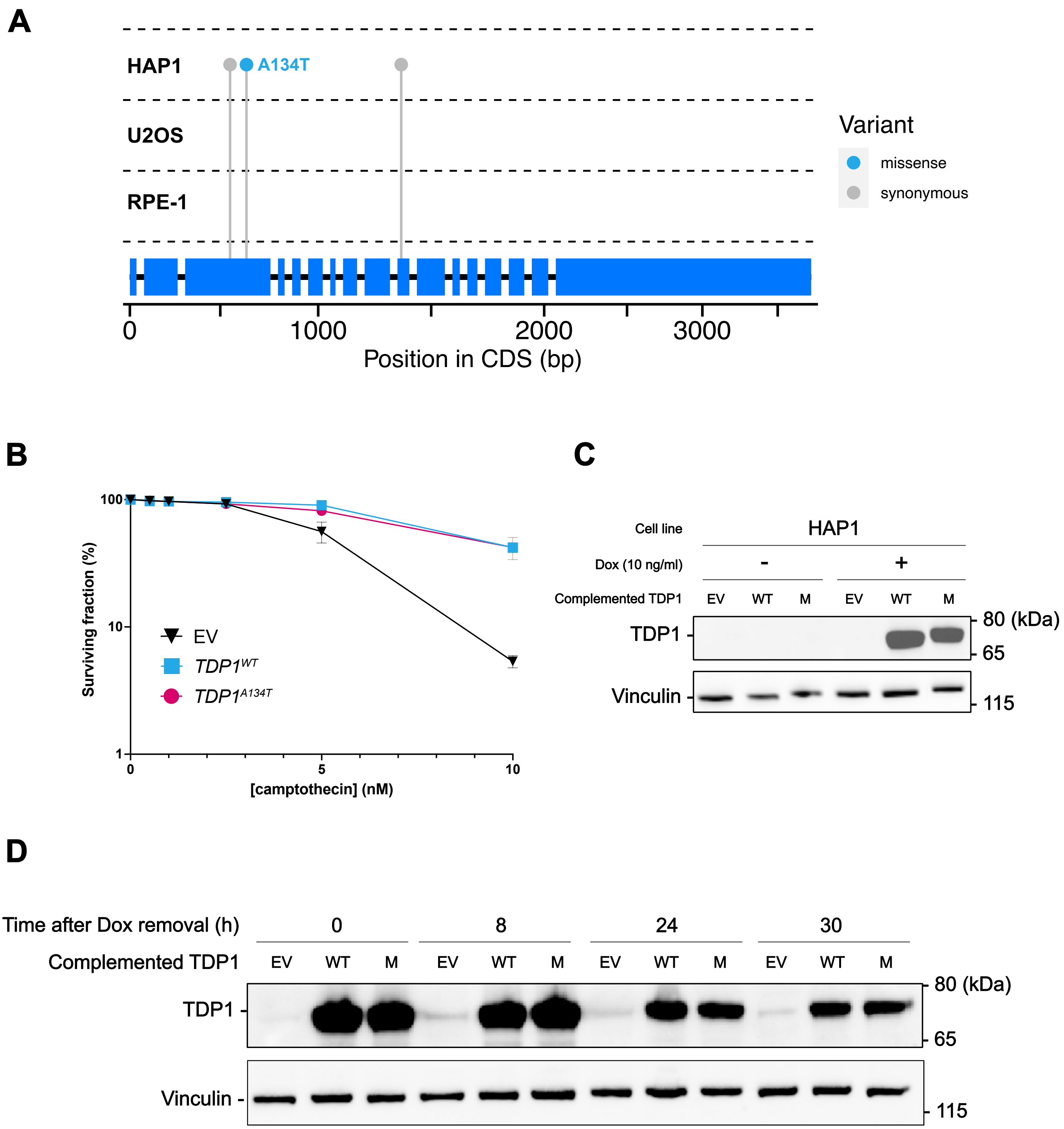
A
HAP1
A134T
U2OS
RPE-1
Variant
missense
synonymous
0
1000
2000
3000
Position in CDS (bp)
B
Surviving fraction (%)
100
10
1
0
5
10
[camptothecin] (nM)
EV
TDP1WT
TDP1A134T
C
Cell line
HAP1
Dox (10 ng/ml)
-
+
Complemented TDP1
EV
WT
M
EV
WT
M
TDP1 -
Vinculin -
80 (kDa)
65
115
D
Time after Dox removal (h)
0
8
24
30
Complemented TDP1
EV
WT
M
EV
WT
M
EV
WT
M
EV
WT
M
TDP1 -
Vinculin -
80 (kDa)
65
115

Supplementary Figure 1. *TDP1_A134T* variant in HAP1 does not affect cellular sensitivity to camptothecin or TDP1 protein stability.

(**A**) Pairwise comparison of the sequenced coding DNA (CDS) of *TDP1* in HAP1, U2OS and RPE-1 cells against the reference genome. *TDP1* variants within the CDS were observed only in HAP1. Introns have been compressed into fixed lengths (50 bp width; black connecting lines) to showcase the entire *TDP1* gene, where the blue horizontal rectangles are individual exons. Genomic variants in the *TDP1* CDS for HAP1 were highlighted as a 'lollipop' and coloured according to the variant type: (●) missense variant and (●) synonymous variant according to the whole-exome sequencing. The only missense variant in HAP1 (A134T) is annotated. (**B**) Cell viability does-response curves of HAP1 cells complemented with empty vector (▼), dox-inducible wild type (■) or A134T (●) TDP1 variant in camptothecin treatment. (**C**) Total cell lysates of cell lines in B (**EV**: empty vector, **WT**: wild type TDP1, **M**: A134T TDP1) were immunoblotted to check for the doxycycline (dox)-induced (10 ng/ml; 24 hours) expression of TDP1. Vinculin serves as a loading control. The sizes (kDa) of reference proteins in PageRuler prestained protein ladder were indicated on the right. (**D**) Total cell lysates of HAP1 *TDP1*$^{-/-}$ complemented with dox-induced wild-type (WT) or A134T (M) TDP1 variants, were immunoblotted for TDP1 protein at 0, 8, 24 and 30 hours after dox removal to probe for protein stability in the cells.

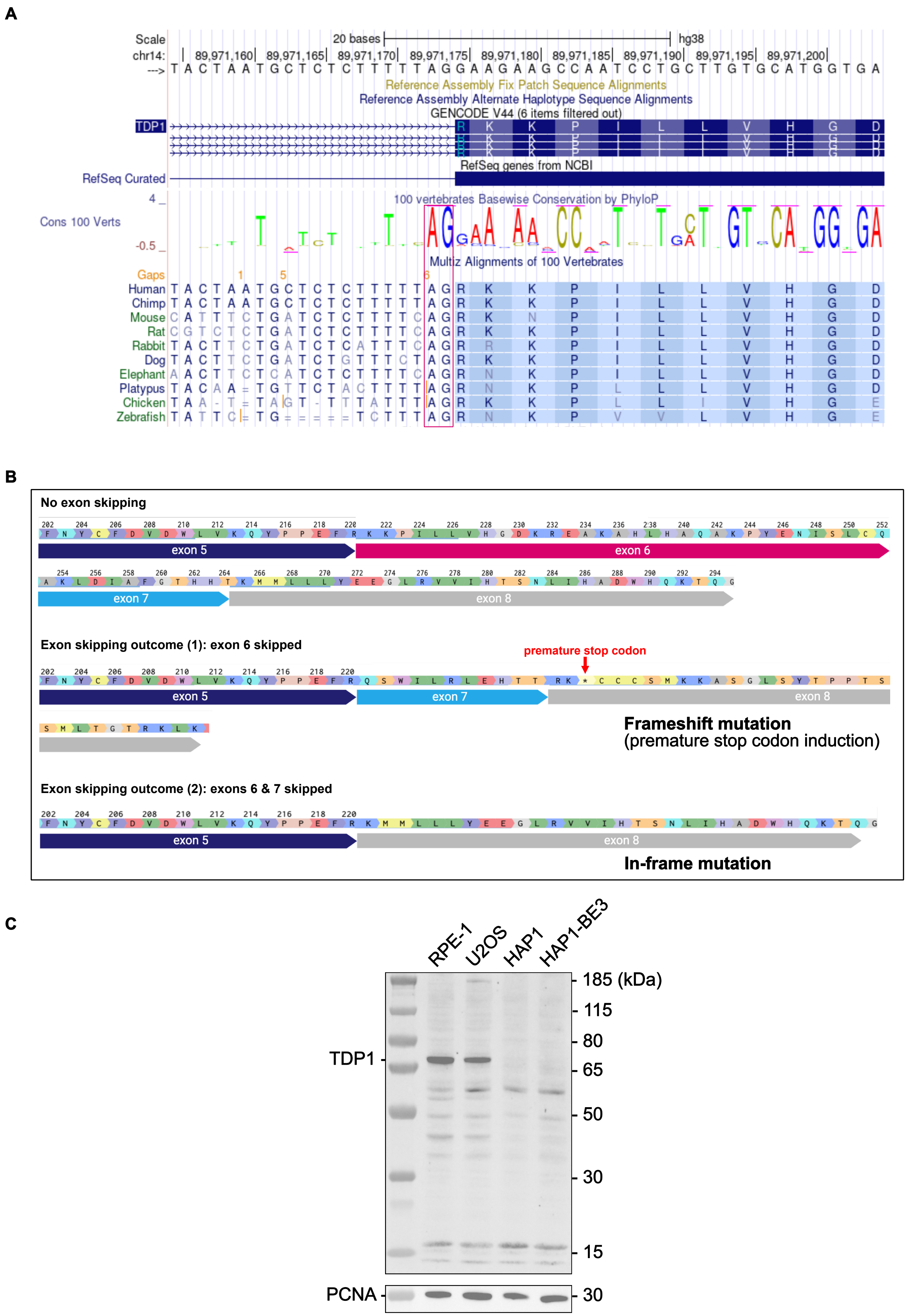
A
Scale
20 bases
hg38
chr14:
89,971,160
89,971,165
89,971,170
89,971,175
89,971,180
89,971,185
89,971,190
89,971,195
89,971,200
Reference Assembly Fix Patch Sequence Alignments
Reference Assembly Alternate Haplotype Sequence Alignments
GENCODE V44 (6 items filtered out)
TDP1
RefSeq genes from NCBI
RefSeq Curated
100 vertebrates Basewise Conservation by PhyloP
Cons 100 Verts
Multiz Alignments of 100 Vertebrates
Gaps
Human
Chimp
Mouse
Rat
Rabbit
Dog
Elephant
Platypus
Chicken
Zebrafish
B
No exon skipping
exon 5
exon 6
exon 7
exon 8
Exon skipping outcome (1): exon 6 skipped
premature stop codon
exon 5
exon 7
exon 8
Frameshift mutation
(premature stop codon induction)
Exon skipping outcome (2): exons 6 & 7 skipped
exon 5
exon 8
In-frame mutation
C
RPE-1
U2OS
HAP1
HAP1-BE3
185 (kDa)
115
80
65
50
30
15
TDP1
PCNA
30

Supplementary Figure 2. *TDP1* splice variant renders TDP1 protein dysfunctional in HAP1 cells.

(**A**) Multiple sequence alignment of *TDP1* across 10 different vertebrates showing the conservation of the splice acceptor site "AG" (in red rectangle) just before exon 6. This is a screenshot from the UCSC human genome browser (GRCh38) with the "100 vertebrates Basewise Conservation by PhyloP" and "Multiz Alignments of 100 Vertebrates" tracks. (**B**) The outcomes on the translated TDP1 protein sequence when erroneous RNA splicing occurs due to the loss of splice acceptor site upstream of *TDP1* exon 6. Two predominant exon skipping outcomes based on sashimi plots (see Figure 2C) and the targeted PCR products of *TDP1* (See Figure 2D) are shown: (1) only exon 6 is skipped (middle; frameshift mutation) or (2) both exons 6 & 7 skipped (bottom; in-frame mutation but a loss of active site H263 in exon 7). For each translated TDP1 protein outcome, the top row features a part of the translated sequence of amino acids with their annotated positions (not labelled after exon skipping) in the protein. The chevrons below are the corresponding exons where each codon of the amino acids is found. The figure is adapted from Benchling. (**C**) The full nitrocellulose membrane immunoblotted for TDP1 as shown in Figure 1B. No distinct band of truncated HAP1-exclusive TDP1 protein (translated from TDP1 mRNA without exon 6 and 7) is observed. PCNA serves as a loading control.

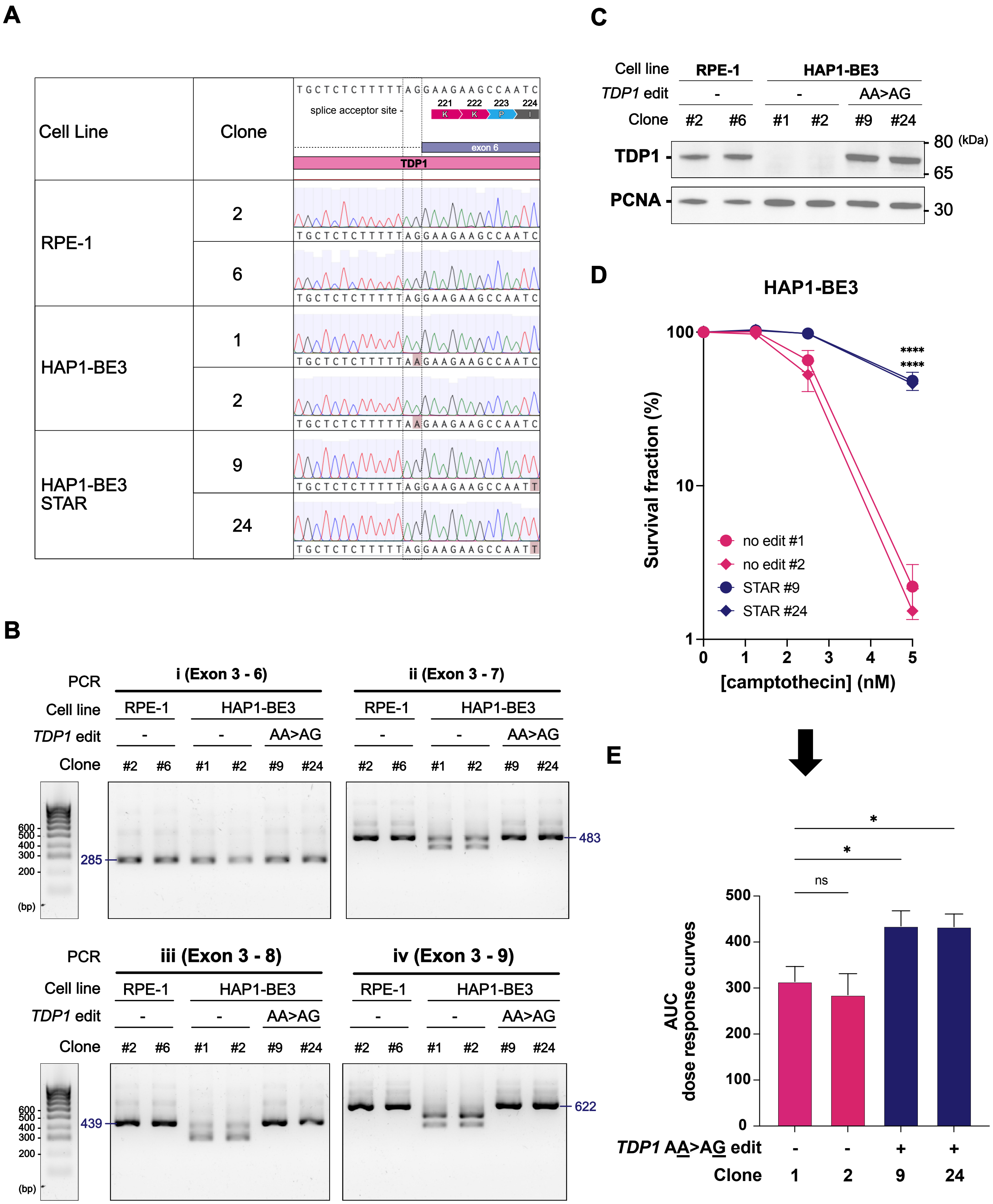
A
Cell Line
Clone
T G C T C T C T T T T T A G G A A G A A G C C A A T C
splice acceptor site
221 222 223 224
K K P I
exon 6
TDP1
RPE-1
2
6
HAP1-BE3
1
2
HAP1-BE3 STAR
9
24
B
PCR
i (Exon 3 - 6)
ii (Exon 3 - 7)
Cell line
RPE-1
HAP1-BE3
TDP1 edit
-
AA>AG
Clone
#2 #6 #1 #2 #9 #24
600
500
400
300
200
(bp)
285
483
iii (Exon 3 - 8)
iv (Exon 3 - 9)
439
622
C
Cell line
RPE-1
HAP1-BE3
TDP1 edit
-
AA>AG
Clone
#2 #6 #1 #2 #9 #24
TDP1
PCNA
80 (kDa)
65
30
D
HAP1-BE3
Survival fraction (%)
100
10
1
0 1 2 3 4 5
[camptothecin] (nM)
****
****
no edit #1
no edit #2
STAR #9
STAR #24
E
AUC dose response curves
500
400
300
200
100
0
ns
*
*
TDP1 AA>AG edit
- - + +
Clone
1 2 9 24

Supplementary Figure 3. Correcting *TDP1* splice-site mutation restores TDP1 protein expression and function in HAP1-BE3 cells.

(**A**) Sanger sequencing chromatograms of two clones each from RPE-1, HAP1 and HAP1-BE3 STAR cells, where the HAP1 STAR clones had had their endogenous *TDP1* splice splice acceptor mutant 'AA' successfully edited into functional 'AG' via DNA-templated homology-directed repair. Two clones (HAP1-BE3 STAR Clone #9 & #24) were identified. A synonymous mutation of *TDP1* c.672C>T was also observed in the edited HAP1-BE3 STAR clones. This mutation was intentionally included in the editing template to minimize the re-annealing of gRNA to the target DNA sequence upon successful genome editing. (**B**) Separation of DNA products amplified using reverse transcribed *TDP1* cDNA in edited (HAP1-BE3 STAR) and unedited HAP1-BE3 clones as well as in RPE-1 clones on a 2% agarose gel to test for *TDP1* mRNA length and purity. The estimated size (bp) of DNA bands without exon skipping (full length of mRNA) for each PCR (i, ii, iii, iv) is annotated. See top part of Figure 2D. The presence of any DNA bands of smaller sizes demonstrate the phenomenon of exon skipping in *TDP1* mRNA. The DNA ladder (left) is made up of DNA bands at 100 base-pair intervals. (**C**) Total cell lysates of HAP1-BE3 STAR and other unedited clones (HAP1-BE3 and RPE-1) were immunoblotted to detect the recovery of TDP1 protein expression upon editing. PCNA serves as a loading control. (**D**) Cell viability dose-response curves to camptothecin of HAP1-BE3 STAR clones relative to unedited HAP1-BE3 clones. 10,000 HAP1-BE3 cells were seeded in technical duplicates 24 hours before camptothecin treatment. Treated cells were incubated for 3 days before measuring cell viability with Alamar Blue fluorescent indicator. Experimental data were the average of 3 biological repeats ± s.e.m. For comparisons against HAP1 no edit clone #1 at 5 nM camptothecin, the *p-values* (**** $p<0.0001$) were calculated using two-way ANOVA. (**E**) Quantified area under curve (AUC) based on the dose-response curves in D. The *p-values* (ns $p>0.05$; * $p<0.05$) were calculated through ordinary one-ANOVA. Despite showing only the comparison of HAP1-BE3 STAR clones #9 and #24 against the HAP1-BE3 no edit clone #1, their comparison against unedited HAP1-BE3 no edit clone #2 were also significant with *p-value*<0.001.

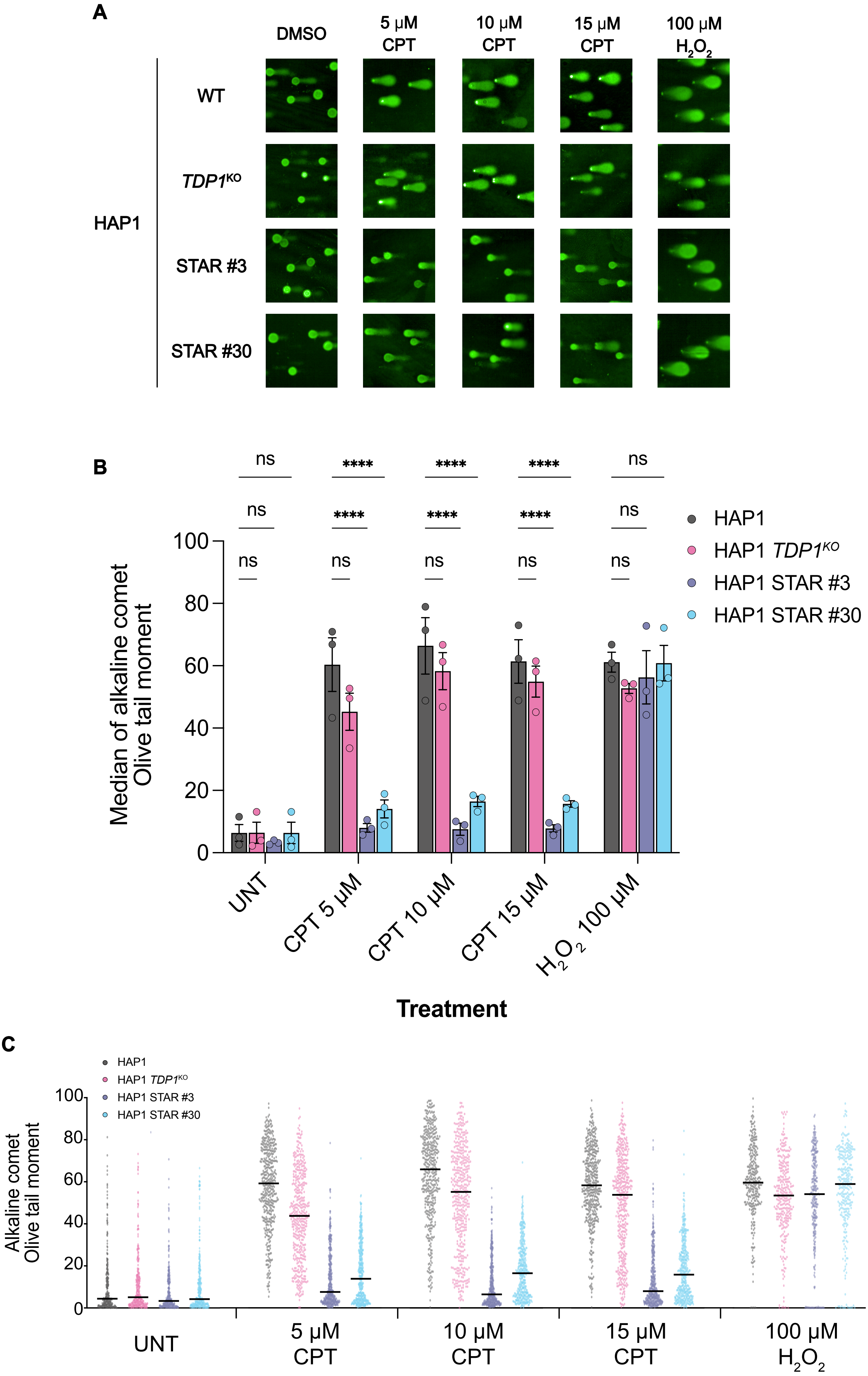
A
DMSO
5 μM CPT
10 μM CPT
15 μM CPT
100 μM H2O2
HAP1
WT
TDP1KO
STAR #3
STAR #30
B
Median of alkaline comet Olive tail moment
Treatment
UNT
CPT 5 μM
CPT 10 μM
CPT 15 μM
H2O2 100 μM
ns
****
HAP1
HAP1 TDP1KO
HAP1 STAR #3
HAP1 STAR #30
C
Alkaline comet Olive tail moment
UNT
5 μM CPT
10 μM CPT
15 μM CPT
100 μM H2O2

Supplementary Figure 4. Alkaline comet assay to determine steady state levels of DNA SSBs upon chemical treatments.

(**A**) Representative images of alkaline comets for (1) “WT” HAP1, (2) HAP1 *TDP1*^{KO}, (3) HAP1 STAR #3 and (4) HAP1 STAR #30 in increasing concentrations of camptothecin. Chemical treatments to induce DNA breaks were carried out for 1 hour before preparing cells for alkaline comet assays. H_2O_2 (100 µM) treatment was included as a positive control for DNA SSBs induction. (**B**) Quantified Olive tail moment of the alkaline comets, which reflects the amount of DNA SSBs. Experimental data were the means (bar height) of the population medians in 3 biological repeats (dots) ± s.e.m. 82-150 comets were analyzed for each condition in each repeat, where the number of analysed comets in a specific condition is kept the same across all cell lines if possible. Different cell lines were compared against “WT” HAP1 in each treatment condition using ordinary two-way ANOVA with the following reported *p-values*: (ns) $p>0.05$ and (****) $p<0.0001$. (A) and (B) are extensions of Figure 3F and 3G respectively with an additional inclusion of camptothecin treatments of 5 and 15 µM. (**C**) Scatter plots showing quantified Olive tail moment of individual alkaline comets for each cell lines under different chemical treatments. Experimental data were the medians (black horizontal line) across 3 biological replicates. 282-450 comets in total (all 3 biological replicates), where the number of analysed comets in a specific condition is kept the same across all cell lines if possible.

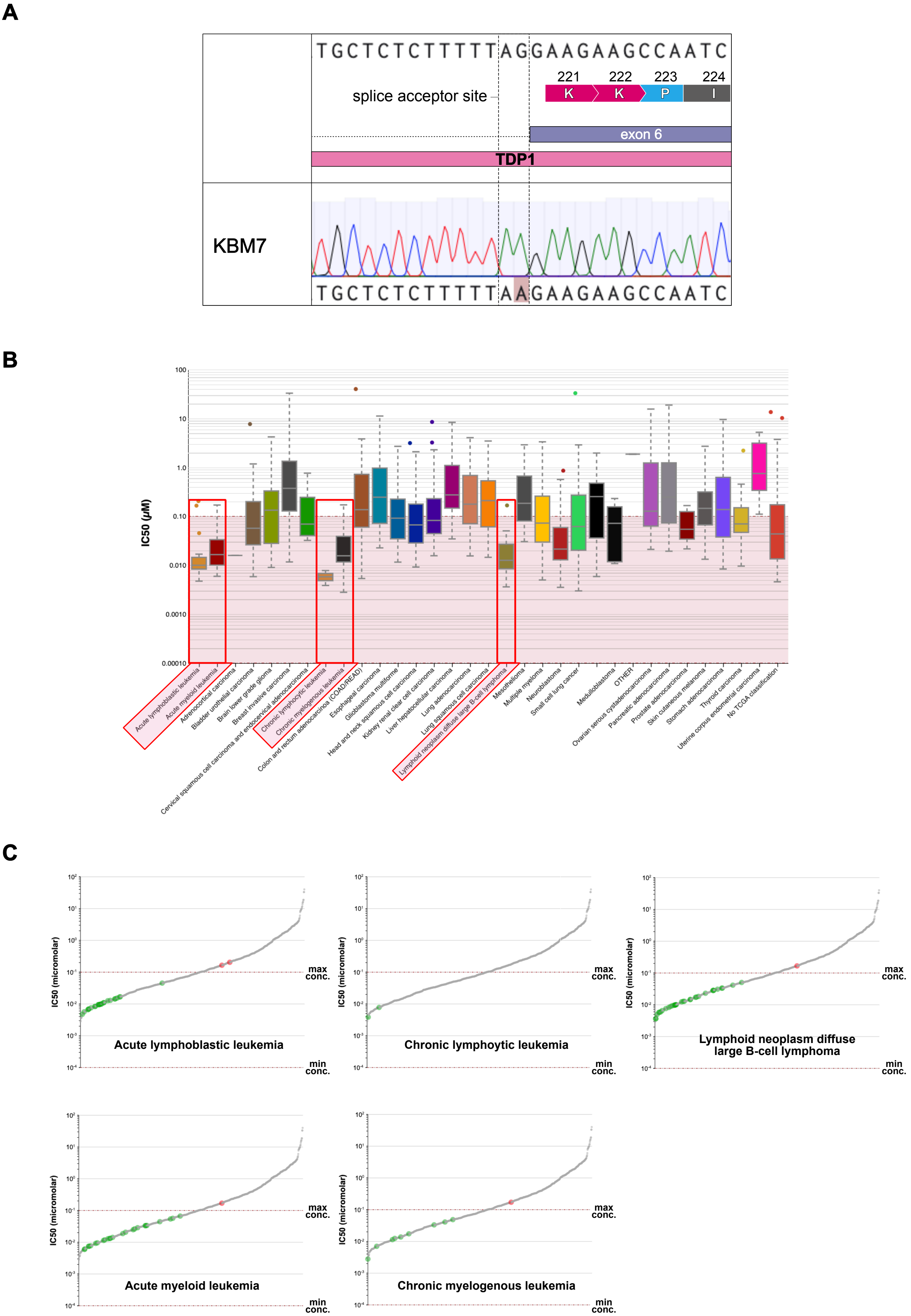

A
TGCTCTCTTTTTAGGAAGAAGCCAATC
221
222
223
224
K
K
P
I
splice acceptor site
exon 6
TDP1
KBM7
TGCTCTCTTTTTAAGAAGAAGCCAATC
B
IC50 (μM)
100
10
1.0
0.10
0.010
0.0010
0.00010
Acute lymphoblastic leukemia
Acute myeloid leukemia
Adrenocortical carcinoma
Bladder urothelial carcinoma
Brain lower grade glioma
Breast invasive carcinoma
Cervical squamous cell carcinoma and endocervical adenocarcinoma
Chronic lymphocytic leukemia
Chronic myelogenous leukemia
Colon and rectum adenocarcinoa (COAD/READ)
Esophageal carcinoma
Glioblastoma multiforme
Head and neck squamous cell carcinoma
Kidney renal clear cell carcinoma
Liver hepatocellular carcinoma
Lung adenocarcinoma
Lung squamous cell carcinoma
Lymphoid neoplasm diffuse large B-cell lymphoma
Mesothelioma
Multiple myeloma
Neuroblastoma
Small cell lung cancer
Medulloblastoma
OTHER
Ovarian serous cystadenocarcinoma
Pancreatic adenocarcinoma
Prostate adenocarcinoma
Skin cutaneous melanoma
Stomach adenocarcinoma
Thyroid carcinoma
Uterine corpus endometrial carcinoma
No TCGA classification
C
IC50 (micromolar)
max conc.
min conc.
Acute lymphoblastic leukemia
Chronic lymphoytic leukemia
Lymphoid neoplasm diffuse large B-cell lymphoma
Acute myeloid leukemia
Chronic myelogenous leukemia

Supplementary Figure 5. *TDP1* splice variant originates from KBM7 and blood cancer cells are generally hypersensitive to camptothecin.

(**A**) Sanger sequencing chromatograms of HAP1's parental cell line, KBM7 showing the genomic sequence of *TDP1* around exon 6. KBM7 cells also possesses the *TDP1* c.660-1G>A splice variant ('AA' splice acceptor site) found in HAP1 cells. (**B**) A snapshot of the half maximal inhibitory concentrations (IC50s; in μM; y-axis) of camptothecin treatment across ~1000 cancer cell lines on the Genomics of Drug Sensitivity in Cancer (GDSC) database, where cell lines of the same tissue types were grouped together in individual boxplots. Blood cancer cells (leukemia and lymphoma) were highlighted in red boxes. (**C**) IC50 values (μM) of camptothecin treatment for individual cancer cells lines (green and red dots, depending on whether it is below or above the threshold) in each of the five tissue types highlighted in (B) and the grey dots representing cancer cell lines of other tissue types.
